# Human placental piwi-interacting RNA transcriptome is characterized by expression from the *DLK1*-*DIO3* imprinted region

**DOI:** 10.1038/s41598-021-93885-3

**Published:** 2021-07-22

**Authors:** Victor D. Martinez, Adam P. Sage, Brenda C. Minatel, Erin A. Marshall, E. Magda Price, Daiana D. Becker-Santos, Wendy P. Robinson, Wan L. Lam

**Affiliations:** 1 British Columbia Cancer Research Centre, 675 West 10th Ave, Vancouver, BC Canada; 2 IWK Health Centre, Halifax, NS Canada; 3 Department of Pathology, Faculty of Medicine, Dalhousie University, 5850/5980 University Avenue, Halifax, NS B3K 6R8 Canada; 4 BC Children’s Hospital Research Institute, Vancouver, BC Canada; 5 Department of Medical Genetics, University of British Columbia, Vancouver, BC Canada

**Keywords:** Germline development, Imprinting, Transcriptomics

## Abstract

The placenta is vital to embryonic development and requires a finely-tuned pattern of gene expression, achieved in part by its unique epigenetic landscape. Piwi-interacting RNAs (piRNAs) are a class of small-non-coding RNA with established roles as epigenetic regulators of gene expression, largely via methylation of targeted DNA sequences. The expression of piRNAs have mainly been described in germ cells, but a fraction have been shown to retain expression in adult somatic tissues. To aid in understanding the contribution of these regulators in the placenta, we provide the first description of the piRNA transcriptome in human placentas. We find 297 piRNAs to be preferentially expressed in the human placenta, a subset of which are expressed at higher levels relative to testes samples. We also observed a large proportion of placental piRNAs to be expressed from a single locus, as distinct from canonical cluster locations associated with transposable element silencing. Finally, we find that 15 of the highest-expressed placental piRNAs maps to the *DLK1-DIO3* locus, suggesting a link to placental biology. Our findings suggest that piRNAs could contribute to the molecular networks defining placental function in humans, and a biological impact of piRNA expression beyond germ cells.

## Introduction

The human placenta is an organ derived from the conceptus that interfaces with maternal tissue to regulate critical aspects of embryonic development and pregnancy, including immune responses, O_2_ and CO_2_ transfer to and from the developing fetus, nutrient delivery, endocrine function, and protection from environmental exposures^1,2^. In order to carry out these highly-specialized functions, gene expression must be tightly regulated, which is largely achieved through the unique epigenetic landscape of the placenta^3,4^. Epigenetic regulation is particularly critical in imprinted regions, of which many have been well-documented to be characteristic to the placenta including the C19MC and C14MC microRNA (miRNA) clusters, the later which lies within the *DLK1-DIO3* region (*14q32.3*)^5–7^. The miRNAs mapped to these imprinted regions are preferentially expressed in the human placenta. However, many of these regions also contain high numbers of other small non-coding RNA (sncRNA) genes, particularly PIWI-interacting RNAs (piRNAs)^8^. While miRNAs have been shown to be involved in key cellular processes in the placenta, piRNAs remain poorly understood in this context despite their intricate evolutionarily-conserved roles in epigenetic regulation and genomic integrity^9,10^.

PiRNAs typically act in the regulation of gene expression through transcriptional silencing via guided DNA methylation^10–13^. Mechanistically, piRNAs form an RNA-induced silencing complex (RISC) with P element–induced wimpy testes (PIWI) proteins, which enables targeting of specific genomic DNA sequences complementary to a given piRNA^14^. As piRNAs predominantly function via DNA methylation, they are critical in the regulation transposable elements (TEs) and non-TE loci as in the case of the imprinted Rasgrf1 locus in the murine male germ line^15^. This accounts for the observation that most of the > 32,000 unique piRNA sequences in humans are expressed and function in germ cells^16^. However, recent evidence indicates that some piRNAs are expressed in somatic tissues, suggesting that their biological importance may extend beyond the germline^17^.

In human germ cells, piRNA-producing loci are mainly grouped in multiple (sometimes hundreds to thousands) discrete genomic locations (piRNA clusters) where long single-stranded piRNA precursor RNAs are transcribed^10,18,19^. These piRNAs are required for post-transcriptional repression/epigenetic silencing of TEs^20^. In contrast, a small proportion of piRNAs derive from single locus located at mRNA coding sequences or 3′ UTRs^18,19^. These sequences constitute ~ 3% of human piRNAs and are expressed in somatic tissues^17^. Due to its origin at gene coding/regulatory sequences, these piRNA sequences could be involved in gene-specific transcriptional control. Thus, the identification of the genomic location of piRNA-producing loci could serve as an indication of their potential molecular roles.

Considering the critical role of piRNAs in epigenetic regulation and the importance of methylation events in the biology of the placenta, we hypothesized that piRNAs exhibit a pattern of expression unique to the placenta and this is not only different from other somatic organs, but also distinct from germ cells. To this end, we used a next-generation sequencing-based approach to elucidate the expression and genomic distribution of piRNAs in the human placenta.

## Results

### piRNA sequences are broadly expressed from the placenta genome

Samples from human placenta (chorionic villi, N = 30) were collected at the BC Children's and Women's Health Centre under relevant ethics approvals. The time from sample collection to RNA extraction varied across samples (processing times are indicated in Supplementary Table 1); however, all samples we immediately put on RNA later after tissue collection, to avoid RNA degradation. After RNA extraction, sequencing of the small RNA transcriptome, and quality processing, reads corresponding to piRNA loci were quantified. The analysis of small RNA sequencing data from these samples revealed a total of 2,037 piRNA sequences with detectable expression (Supplemental Table 2).

Mapping of the 2,037 piRNA sequences to their genomic locations revealed that specific chromosomes contain a high number of loci harbouring piRNA sequences, namely chromosomes 6 (90 loci), 16 (67 loci), 14 (58 loci), and 1 (53 loci). These loci are indicated in Supplemental Table 2. However, some of the piRNAs associated with these chromosomes mapped to one or more additional locations in the human genome, complicating the assignment of some of piRNAs to a unique location. Notably, 19 placenta-expressed piRNAs mapped to the mitochondria, representing 45% of the 42 known piRNA loci mapped to the mitochondria genome. To focus on the sequences most consistently expressed in these samples, we applied the following filtering criteria: (i) ≥ 10 reads detected across all samples and (ii) normalized expression levels ≥ 1 RPM in 10% of the samples. This process resulted in the identification of a set of 297 piRNAs sequences distributed across the human placental genome (Supplemental Table 3). Once we restricted our analysis to (i) those piRNA that can be confidently mapped to a single location and (ii) those that meet the expression cut-offs mentioned above, a cluster of piRNA harboured in chromosome 14 emerged as a piRNA gene cluster prevalently expressed in the placenta.

### piRNAs display a unique expression pattern in placental tissues

**Figure 1:**
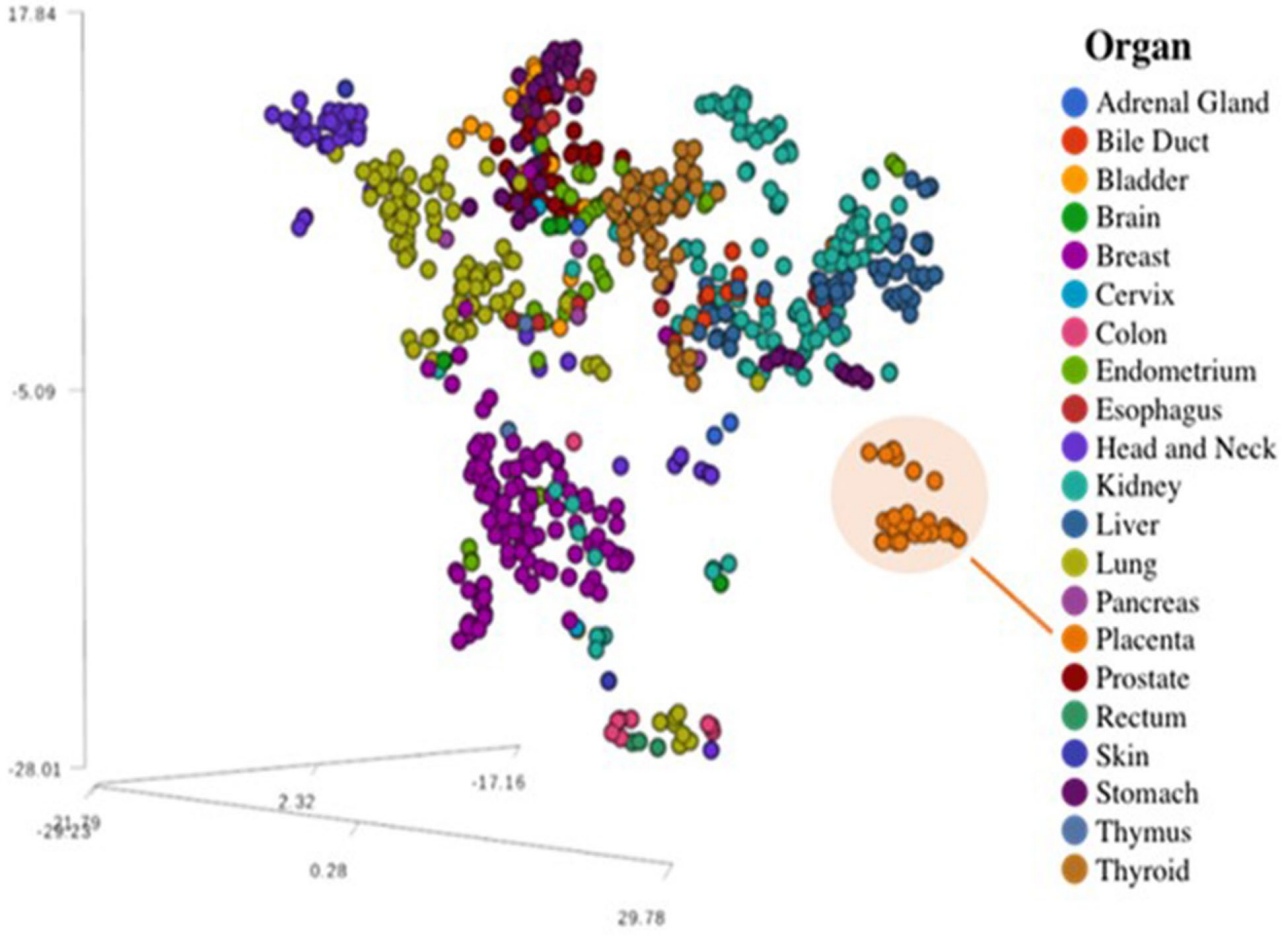
Expression of piRNAs distinguishes human placenta from adult tissue. A tSNE plot displaying samples clustered based on the combined expression of the 297 piRNAs identified in placenta and their levels in other adult tissues. Human placenta samples are highlighted in an orange circle. In this t-SNE dimensionality reduction representation, the expression of the 297 piRNAs (i.e., 297 dimensions) has been reduced to three dimensions (represented by the 3 axes). The numbers in the axes are measurements of the pairwise distances calculations for a given reduced space.

We next sought to assess whether these sequences were preferentially expressed in the placenta relative to other somatic tissue types. In addition to the expression of individual piRNA transcripts, we assessed the combined expression of piRNAs to identify unique features in the placenta. We examined the expression levels of the 297 placenta-derived piRNAs in 675 samples from 18 human adult non-malignant tissue types obtained from The Cancer Genome Atlas (TCGA). Using the same analysis pipeline described above, we determined the per-sample RPM expression of these 297 piRNAs across human tissues (Supplemental Table 3). The results revealed 11 piRNAs that were exclusively expressed in placenta samples, while 53 were expressed across all tissues studied. Remarkably, the combined expression pattern of the 297 placenta-derived piRNAs was able to distinguish the placenta samples from all other organs (Fig. 1).

### The piRNA expression pattern in placenta differs from the testis

We compared the expression of the 297 placental piRNAs with those expressed in testes samples. To perform this analysis, we used the same pipeline as described for the placenta samples to examine sncRNA sequencing data from human testis samples (n = 3, NCBI BioProject: PRJNA196749)^21^. This process revealed 5567 piRNAs expressed in testes (≥ 10 reads; RPM ≥ 1). Of the 297 placenta piRNAs, 187 met the same expression criteria in testes. Many of these piRNAs were strongly differentially expressed between the two contexts, wherein 95 piRNAs showed five-fold or higher median expression in testes, while 22 piRNAs display five-fold or higher median expression in placenta samples (Supplementary Table 4). Additionally, five placental piRNAs were not detected in any of the testes samples (piR-hsa-24143; piR-hsa-26947; piR-hsa-27513; piR-hsa-30734; piR-hsa-32299). This placental-specific expression pattern suggests potential functions for piRNAs extending beyond known functions of piRNA in germ cells.

### Placenta-derived piRNA are primarily derived from single loci

**Figure 2:**
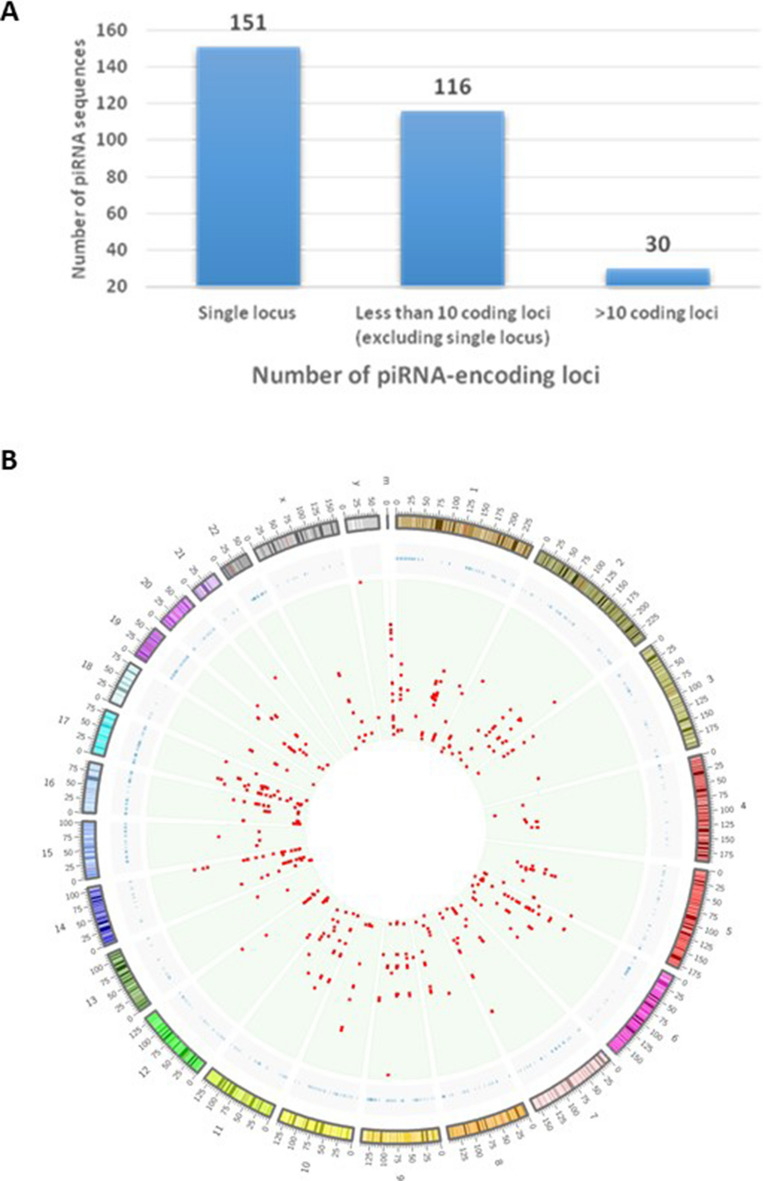
Genome-wide distribution of regions harbouring piRNA in the human placenta. **(A)** The bar graph depicts the number of piRNAs (y-axis) transcribed from 1, < 10, and > 10 genomic loci (x-axis). More than half of the piRNAs expressed in placenta (n = 151/297) are transcribed from a single locus, while 89.6% (n = 266) of the expressed sequences are transcribed from less than 10. **(B)** Circular representation of the human genome (circos plot) highlighting the chromosomal location of the 636 loci encoding the 297 placental-expressed piRNAs. The outer circle represents human chromosomes (1–22, X, Y and M, hg19 coordinates). The position of human piRNA clusters are shown in the concentric blue layer interior to the chromosomal positions. Each red dot in the green concentric circle represents one of the 636 piRNA loci.

As the organization of genomic regions where piRNA are located can be suggestive of potential function, we then focused our attention on the genomic localization of the 297 placental piRNAs. While individual human piRNAs typically originate from hundreds of genomic loci, among the 297 placental piRNAs only 30 (10.4%) are transcribed from > 100 loci distributed across the genome. In fact, more than half of the piRNAs (151/297) expressed in the placenta are transcribed from a single locus, and the remaining 116 map to fewer than 10 genomic loci (Fig. 2A). Overall, these 267 (90%) piRNAs map to only 636 chromosomal locations (Fig. 2B). The under-representation of cluster-derived piRNAs in the placenta is further evidenced by our observation that none of the 297 placental piRNAs map to known human piRNA cluster locations^22^. As cluster-derived piRNAs predominantly act in the silencing of TEs, the high proportion of single-locus piRNAs indicates that placenta-piRNAs are likely involved in other genomic activities.

### Single-loci piRNAs are enriched in the DLK1-DIO3 locus

**Figure 3:**
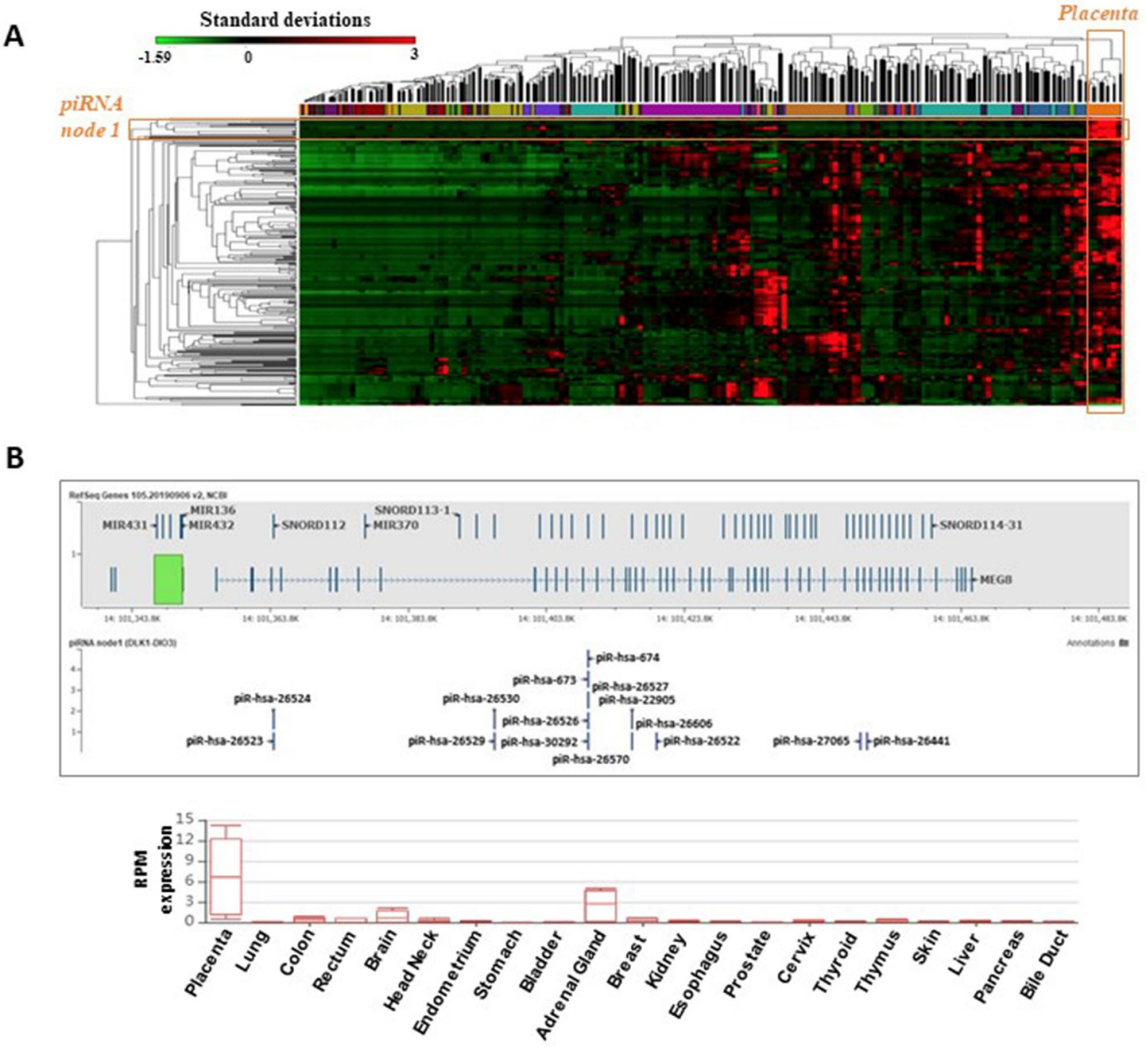
Expression of the 297 piRNAs in the placenta vs human somatic tissues. **(A)** An unsupervised hierarchical clustering of samples based on the expression of the 297 piRNAs expressed in placenta samples and their corresponding expression in human adult tissues. The analysis indicates that most of the identified piRNAs display higher expression in the placenta compared with other adult somatic tissues. **(B)** Comparison of the median expression of the 15 piRNAs from node 1 across human placenta and somatic tissues. The expression range (represented by a whisker plot with the median, 25th and 75th percentiles and maximum and minimum values) of the 15 piRNAs from node 1 across human placenta and somatic tissue (tissues described in the X-axis).

Genomic imprinting is a predominant epigenetic feature that controls the expression of critical genes in the placenta, including numerous miRNAs^23^. To determine if genomic imprinting plays a role in the expression of placental piRNAs, we first performed an unsupervised hierarchical clustering analysis of piRNAs expressed across all samples to identify piRNAs highly expressed in the placenta but lowly or not expressed in other organs (Fig. 3A). Overall, the expression of these piRNAs was highest in placenta samples, distinguishing the placenta from other tissues in the analysis. The analysis also revealed a set of 16 piRNAs (node 1 in Fig. 3A) which was highly and nearly exclusively expressed in the placenta. Of particular interest is the observation that 15 of the 16 piRNAs in node 1 are single-locus piRNAs, which all map within the *Maternally Expressed 8* (*MEG8*) gene that resides within the *DLK1-DIO3* (*14q32.31*) imprinted locus. While the 15 piRNAs were not exclusively expressed in the placenta (with detectable levels in other tissues, particularly in adrenal gland as well as fetal and adult brain tissue), their combined expression pattern is a distinguishing feature in the placenta samples (Fig. 3B, Supplementary Table 5).

## Discussion

In this study, we present the first comprehensive description of the human placenta piRNA transcriptome. The detection of over 2000 piRNA sequences and further characterization of 297 sequences consistently expressed in our cohort (n = 30) is a significant expansion of the known small RNA transcriptome in this organ and represents a resource for further elucidation of the functional impact of piRNA expression in the placenta. We find that not only is a substantial proportion of piRNAs expressed in human placenta samples, but their expression is able to distinguish the placenta from other adult (non-cancerous) tissues. While the differences observed amongst placenta and other tissues were deduced from normalized expression data generated through the same platform, we cannot rule out the influence of technical confounding factors as additional sources of variation.

The majority of piRNAs expressed in the placenta are transcribed from a single genomic locus. Single-locus piRNAs are typically involved in the regulation of gene expression and the maintenance of specific molecular features, which may impact placenta-specific biology. These observations deviate from the established roles for piRNAs in the transcriptional silencing of TEs, which is the typical activity for cluster-derived piRNAs. Further, we did not observe any of the placental piRNAs to be derived from known piRNA cluster locations. Together, these results suggest that a fraction of human piRNAs remain active in the placenta, where they could participate in other less common piRNA-related functions, such as gene-specific regulation and mediate placenta-specific epigenetic regulation.

We identified that the placenta displays a specific piRNA expression pattern compared to other somatic tissues. Remarkably, the subset of piRNAs exhibiting the highest differences in expression compared to other tissues map to the *DLK1-DIO3* imprinted locus, particularly within the *MEG8* gene. *MEG8* is a non-coding transcript that is preferentially expressed from the maternal allele and is known to host other classes of sncRNAs^7^. Moreover, the conserved chromosomal region in the *DLK1-DIO3* imprinted domain also contains one of the largest miRNA clusters in the human genome (C14MC), and its expression is regulated by an intergenic germline-derived differentially-methylated region located upstream from the miRNA cluster^23^. As several miRNAs from this cluster have been associated with pregnancy and other placenta-specific functions^5^, it can be suggested that piRNAs transcribed from this region may also participate in similar processes. The elucidation of the role of these piRNAs will require additional studies, likely in animal models. It is important to note that, while some of the mechanisms regulating piRNA expression in other species (e.g. mouse) are similar, the primary sequence of piRNAs is poorly conserved (even for syntenic to those in other placental mammals)^10,18^.

The genomic location of some of the 15 piRNAs included in the 14q cluster partially overlaps with small nucleolar RNAs (snoRNAs), which are contained in intronic segments of the long non-coding RNA MEG8. SnoRNAs harbouring piRNAs involved in gene expression regulation has been previously described. The GAS5 long non-coding RNA is known to host snoRNAs, which can generate piRNAs capable of inducing specific gene expression activation^24^. Similarly, highly expressed mouse piRNAs displaying cell type specificity map to an intronic region of the Rab26os gene which also locates a snoRNA^25^. Furthermore, it has been shown that snoRNA-derived piRNA can induce decay of the human IL-4 pre-mRNA (via sequence complementarity binding), downregulating the protein levels^26^. While the precise mechanisms of generation of snoRNA-derived piRNAs has not been yet elucidated, a plausible possibility is that snoRNAs can be processed to piRNAs and/or other small-noncoding RNAs and participate in regulation of gene expression^27^.

The identification of 19 piRNAs mapped to the mitochondrial genome that are expressed in the human placenta is also of interest given the unique role of placental mitochondria. Mitochondria are multifunctional organelles that undergo rapid changes in response to energy needs, and play an important role in regulating nutritional delivery to the fetus^28,29^. In the syncytial trophoblast of the placenta, mitochondria undergo extensive remodeling, becoming fragmented, smaller and denser; correspondingly metabolic activity is suppressed, and these mitochondria are transformed to instead produce progesterone, a critical hormone in pregnancy^30^. As the processes occurring in the placenta are characterized by high energy requirements, the expression of piRNAs mapped to the mitochondrial genome warrants further investigation.

Together, our results not only emphasize the gene regulatory potential of piRNAs preferentially expressed in the human placenta, but also highlight the important biological information encoded within the small non-coding RNA transcriptome. Our work will provide a foundation for further mechanistic evaluation of piRNAs in somatic tissues. Future analyses may seek to determine the biological effects that are associated with the expression of these placental piRNAs, through DNA-target identification and analysis of transcripts with aberrant expression patterns in different contexts. Thus, the study of the expression of piRNAs presents a valuable resource to understand intricate mechanisms of gene regulation in previously uncharted genomic landscapes.

## Methods

### Sample collection and processing

All placenta samples (chorionic villi) (N = 30) were collected at the BC Children's and Women's Health Centre, Vancouver, BC. All experimental protocols were approved by the University of British Columbia/Children’s and Women’s Health Centre of British Columbia Research Ethics board (H16-02280). These samples were collected as previously reported and used in a deidentified manner for the present study^31^. The information for these samples was described in Supplemental Table 1. Briefly, chorionic villi samples of approximately ~ 0.5 cm^3^ were collected from the fetal side of the placenta to avoid the possibility of contamination with the maternal decidua. Each piece was thoroughly washed with 1× PBS until all traces of visible blood were eliminated before preservation in a 5 ml tube containing 1 ml of RNAlater (Invitrogen). Processing time after delivery ranged from one to 192 h. The samples were left at 4 °C overnight and then transferred to labelled cryovials to be stored at -80 °C until used. The RNAlater was removed before starting the RNA extraction procedure. Samples were homogenized with 1 ml of TRIzol reagent (ThermoFisher Scientific, USA) following the vendor’s specifications. Genomic DNA was removed using the RNase-Free DNase Set (Qiagen, Germany). RNA quality was assayed on an Agilent Bioanalyzer 2100 (Agilent, USA). Prior to sequencing, small RNA fractions were depleted of ribosomal RNA by hybridization, using the NEBNext rRNA Depletion Kit (New England BioLabs, USA).

Any case with known chromosome abnormalities was excluded and remaining samples were screened for large changes using multiplexed ligation-dependent probe amplification (MLPA) of subtelomeric probes on each chromosome (SALSA MLPA Subtelomeres Mix, MRC-Holland). Clinical information, including gestational age at delivery, sex, and inferred MLPA genotype is provided in Supplemental Table 1. RNAlater was removed before starting the RNA extraction procedure. Samples were homogenized with 1 ml of TRIzol reagent (ThermoFisher Scientific, USA) following the vendor’s specifications. Genomic DNA was removed using the RNase-Free DNase Set (Qiagen, Germany). RNA quality was assayed on an Agilent Bioanalyzer 2100 (Agilent, USA). Prior to sequencing, small RNA fractions were depleted of ribosomal RNA by hybridization, using the NEBNext rRNA Depletion Kit (New England BioLabs, USA). The possibility that some preterm samples would have developed observable placental or fetal dysfunctions had the pregnancies progressed further cannot be excluded. However, the absence of outliers in hierarchical cluster and t-SNE analyses suggest that the samples considered in the analysis have a similar expression pattern of piRNAs at the time of sampling.

All experiments were performed in accordance with relevant named guidelines and regulations.

### Sequencing

**Figure 4:**
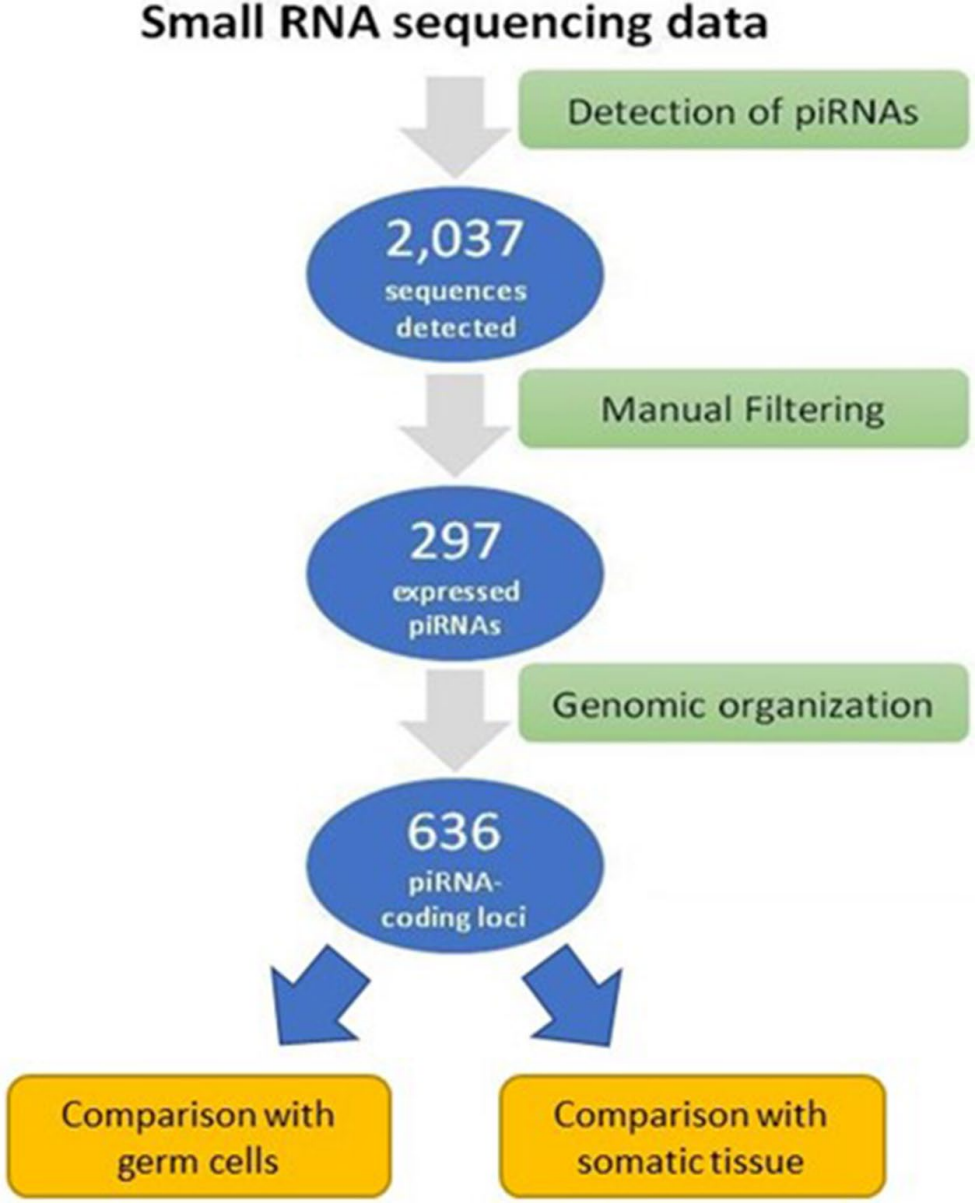
Characterization of the placenta piRNA transcriptome. Small RNA sequence data derived from 30 human placenta samples was processed for the quantification of annotated piRNA sequences. Raw sequencing reads were subject to a standard quality control process to discard: (i) adapters, (ii) reads with a length < 16 nt and (iii) reads with a quality level (Phred) < 20. High-quality reads (in fastq format) were used as input for the miRmaster small non-coding RNA analysis platform^34^. Reads mapping to a piRNA annotation model (hg19) available from piRBase (http://regulatoryrna.org/database/piRNA/index.html) were quantified, resulting in 2,037 sequences that matched the genomic coordinates of annotated human piRNAs. This subset of sequences was further filtered to identify the set of more robustly expressed sequences by (i) considering piRNA species covered by ≥ 10 reads and (ii) piRNAs with normalized expression levels (RPM) ≥  1 in 3 or more samples. As a result, we obtained a set of 297 piRNA showing consistent expression across the placenta samples. These 297 piRNA sequences were transcribed from 636 loci (some piRNA were transcribed from multiple loci). The same process was followed for testis and somatic samples. Only the expression of the 297 piRNAs preferentially expressed in placenta was compared with this two group of samples (testes and somatic tissue).

Total RNA extracted from placenta samples was used for small RNA sequencing library construction at the Canada's Michael Smith Genome Science Center (GSC, Vancouver, Canada). Briefly, RNA quality was checked using Agilent Bioanalyzer RNA nanochip. Samples that passed the quality check were arrayed into a 96-well plate (1 μg of total RNA was used for each sample) and sequenced using the Illumina HiSeq 2000 platform. Raw sequencing reads were subject to a quality control process to discard: (i) adapters, (ii) reads with a length < 16 nt and (iii) reads with a quality level (Phred) < 20. High-quality reads (in fastq format) were aligned to the NCBI GRCh37 reference human genome build with the STAR aligner (v 2.4.1d) using the default parameters^32^. Aligned reads (BAM files) were quantified using the PartekFlow platform as reads per kilobase per million (RPKM). The methods used for sample accrual, sequencing library construction, and data analysis are detailed in Fig. 4. Sequencing data from the human placenta samples has been deposited in the Gene Expression Omnibus (GEO, accession number: GSE164178).

The somatic tissues used in this study were obtained from The Cancer Genome Atlas (TCGA) repository (data accessed through dbgap Project ID: 6208). These samples were sequenced at the Canada's Michael Smith Genome Science Center which generated sequence data for the TCGA project. In order to make the data as comparable as possible, our placenta samples were subject to the same library preparation, sequencing (including pooling strategy) and data quality protocols. The raw sequencing data (fastq files) of the TCGA samples were processed using the same bioinformatics pipeline used for the placenta samples.

Our sequencing protocols, as well as the one used for somatic tissues, considered the generation of reads with an average size over 16 and below 40 nt. While this was not expected to completely abrogate the possibility of the detection of transcripts going across piRNA loci, it had greatly enriched for miRNA and piRNAs. To exclude sequences mapping to miRNA loci, we also discarded reads with a length that will likely correspond to a miRNA (below 23 nt). To discard potential RNA fragments originated from other biological processes (e.g. degradation), we considered those piRNA candidate sequences that were identified systematically across samples (both in somatic and placenta tissues), and under the assumption that these fragments would be randomly generated and observation of the same random fragment across several independent samples would be a less likely event. Finally, localization and mapping of the reads assigned to piRNAs was performed using human-specific piRNA genomic coordinates determined by functional studies and that are publicly available^16,33^.

### piRNA quantification

piRNA quantification was performed using PartekFlow (Partek Inc., MO, USA) and the mirmaster platform^34^. In PartekFlow, reads were assigned to piRNA genomic loci, according to the coordinates listed in pirBase, available at http://regulatoryrna.org/database/piRNA/index.html^10,13,16,33^. The relative quantity of reads was scaled using the Reads per million mapped reads (RPM) method. The detailed workflow for identification of expressed piRNA in placenta and comparison with the corresponding expression in testes and somatic tissues is shown in Fig. 4.

### t-distributed stochastic neighbor embedding (t-SNE)

The t-SNE technique was used for identifying similarities across the patterns defined by the expression of piRNAs and to reduce data dimensionality of the sncRNA expression datasets from the placenta and somatic tissues. Briefly, this method gave each sample a location in a three-dimensional map, and cluster samples based on similar overall sncRNA expression pattern. This technique was optimized to allow the implicit structure of the dataset to influence the way in which a subset of the data is displayed. The analysis was performed in the t-SNE implementation in our PartekFlow platform.

## Supplementary Information

Supplementary Legends.

Supplementary Figure 1.

Supplementary Table 1.

Supplementary Table 2.

Supplementary Table 3.

Supplementary Table 4.

Supplementary Table 5.
