# Supplementary material for: Human placental piwi-interacting RNA transcriptome is characterized by expression from the DLK1-DIO3 imprinted region
Source: Sci Rep. 2021 Jul 22;11:14981. doi: 10.1038/s41598-021-93885-3 (PMC8298716; doi:10.1038/s41598-021-93885-3)
Supplement: Supplementary file 1 — Supplementary Legends. [file 41598_2021_93885_MOESM1_ESM.docx]

**List of supplemental data**

- Supplemental Table 1 – Sample information
- Supplemental Table 2 – piRNA expression for each placenta sample
- Supplemental Table 3 – 297 piRNAs, expression per sample
- Supplemental Table 4 – Comparison of expression levels between placenta and germ cells
- Supplemental Table 5 – Expression of the piRNAs transcribed from the DLK1-DIO3 imprinted region in placenta and fetal brain
- Supplemental Figure 1 - Similarly Matrix comparing the expression of the 297 piRNA preferentially expressed in placenta with their corresponding expression in fetal brain samples.

**Supplementary Figures Legend**

**Supplement Figure 1:** Similarly Matrix comparing the expression of the 297 piRNA preferentially expressed in placenta with their corresponding expression in fetal brain samples. The correlation between samples was measured using the spearman rank correlation method. Correlation values are displayed in the upper bar and range from -1 (blue, low correlation) to 1 (red, high correlation). The diagonal high correlation line represent the comparison of each sample to itself, which is denoted by the highest correlation value (value = 1)
