# Supplementary figures and images for: Human placental piwi-interacting RNA transcriptome is characterized by expression from the DLK1-DIO3 imprinted region
Source: Sci Rep. 2021 Jul 22;11:14981. doi: 10.1038/s41598-021-93885-3 (PMC8298716; doi:10.1038/s41598-021-93885-3)

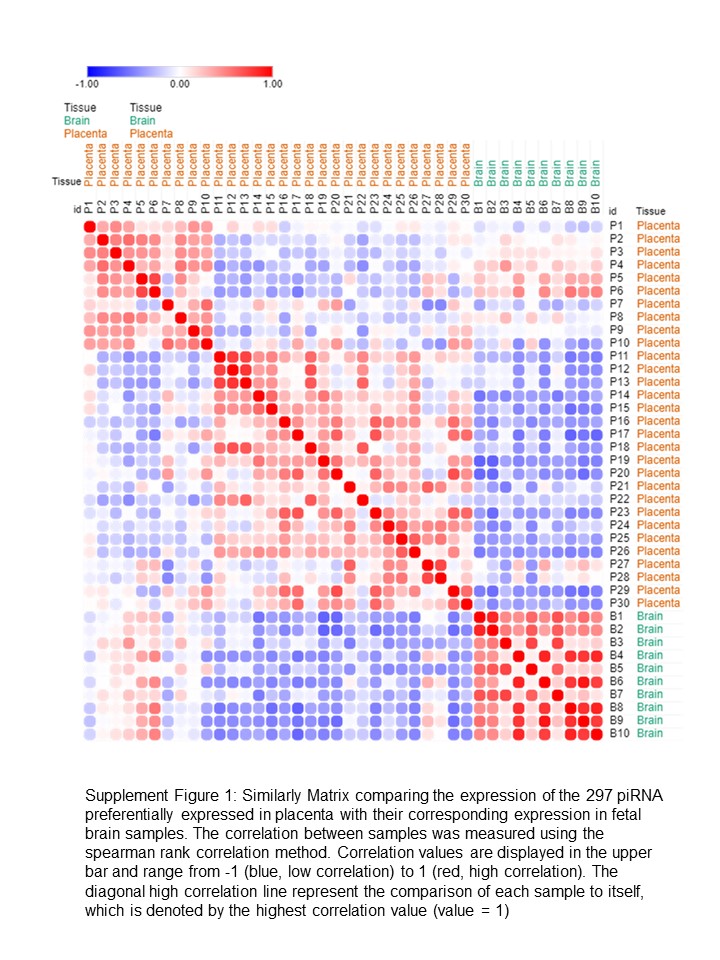

Supplement: Supplementary file 2 — Supplementary Figure 1. [file 41598_2021_93885_MOESM2_ESM.jpg]
